# Immune checkpoint inhibitors as the second-line treatment for advanced esophageal squamous cell carcinoma: a cost-effectiveness analysis based on network meta-analysis

**DOI:** 10.1186/s12885-024-12423-2

**Published:** 2024-05-29

**Authors:** Xiuli Yang, Xiaochun Zheng, Sang Hu, Jinlong Huang, Miaomiao Zhang, Ping Huang, Jiangfeng Wang

**Affiliations:** 1 Center for Clinical Pharmacy, Cancer Center, Department of Pharmacy, Zhejiang Provincial People’s Hospital (Affiliated People’s Hospital), Hangzhou Medical College, 158 Shangtang Rd, Gongsu District, Hangzhou, Zhejiang 310014 China; 2 Central Hospital of Haining, Jiaxing, Zhejiang China; 3 School of Pharmacy, Hangzhou Normal University, Hangzhou, Zhejiang China; 4 Department of Pharmaceutical Services, Ipharmacare Ltd, 2073 Jinchang Rd, Yuhang District, Hangzhou, 310030 Zhejiang China

**Keywords:** Cost-effectiveness, Network meta-analysis, Immune checkpoint inhibitors, Esophageal squamous cell carcinoma, Sintilimab, Tislelizumab, Camrelizumab, Nivolumab, Pembrolizumab

## Abstract

**Background:**

Immune checkpoint inhibitors (ICIs) have demonstrated superior clinical efficacy in prolonging overall survival (OS) as the second-line treatment for advanced or metastatic esophageal squamous cell carcinoma (ESCC), and were recommended by the guidelines. However, it remains uncertain which ICI is the most cost-effective. This study assessed the cost-effectiveness of ICIs as the second-line treatment for ESCC based on the perspective of the Chinese healthcare system.

**Methods:**

A network meta-analysis (NMA) was performed to obtain the Hazard ratios (HRs) for indirect comparisons. A three-state Markov model with a 10-year time horizon was conducted to assess the cost-effectiveness. The state transition probabilities were calculated with Kaplan-Meier (KM) curves data from clinical trial and HRs from the NMA. Utilities and costs were derived from local charges or previously published studies. Univariate and probabilistic sensitivity analyses (PSA) were performed to examine model robustness. The results were assessed based on the total costs, quality-adjusted life years (QALYs), and incremental cost-effectiveness ratios (ICERs).

**Results:**

Five clinical trials (ATTRACTION-3, ESCORT, KEYNOTE-181, ORIENT-2, RATIONALE-302) with a total of 1797 patients were included in the NMA. The NMA showed that both camrelizumab and tislelizumab received relatively high rankings for progression-free survival (PFS) and OS. Compared with sintilimab, treatment with tislelizumab and camrelizumab gained 0.018 and 0.034 additional QALYs, resulting in incremental ICERs of $75,472.65/QALY and $175,681.9/QALY, respectively. Nivolumab and pembrolizumab produced lower QALYs and greater costs, suggesting that both were dominated in comparison to sintilimab. HRs and health state utilities were the most influential parameters in most univariate sensitivity analyses of paired comparisons. PSA results suggested that sintilimab had an 84.4% chance of being the most cost-effective treatment regimen at the WTP threshold of $38,223.34/QALY. In the scenario analysis, sintilimab would no longer be cost-effective, if the price of camrelizumab was assumed to decrease by 64.6% or the price of tislelizumab was assumed to decrease by 16.9%.

**Conclusions and relevance:**

Among the five potential competing ICIs, sintilimab was likely to be the most cost-effective regimen as the second-line treatment for locally advanced or metastatic ESCC in China.

**Supplementary Information:**

The online version contains supplementary material available at 10.1186/s12885-024-12423-2.

## Key points

**Question:**

Which immune checkpoint inhibitor (ICI) is the most cost-effective for the second-line treatment of the patients with metastatic esophageal squamous cell carcinoma (ESCC)?

**Findings:**

At a willingness-to-pay threshold of $38,223.34/QALY, sintilimab was cost-effective in 84.4% of the simulations, the highest of the ICIs.

**Meaning:**

Sintilimab was the most cost-effective option compared to other ICIs as the second-line therapy for ESCC in China.

**Supplementary Information:**

The online version contains supplementary material available at 10.1186/s12885-024-12423-2.

## Introduction

Esophageal cancer is a severe malignancy that develops from the esophageal epithelium. It was the seventh most common cancer and the fifth leading cause of cancer mortality worldwide [1]. Eastern Asia had the greatest incidence rates of esophageal cancer, due to the massive burden in China [1]. In 2016, it was predicted that 252,500 new cases of esophageal cancer were discovered and that 193,900 patients died as a result of esophageal cancer in China [2]. According to histological type, esophageal cancer is mainly categorized into squamous cell carcinoma and adenocarcinoma. Esophageal squamous cell carcinoma (ESCC) accounts for more than 85% of all esophageal cancers [3]. In addition, 68.7% of all ESCC cases worldwide occurred in China [4]. Esophageal cancer has a marked aggressiveness. The prognosis of esophageal cancer mainly depends on the local infiltration and distant metastasis. Regrettably, most esophageal cancer patients presented with an advanced stage at the time of their initial diagnosis, and the five-year survival rate among Chinese esophageal cancer patients was approximately 18.4% [5]. Fortunately, developing new treatments could potentially lead to greater clinical benefits for ESCC patients.

Immune checkpoint inhibitors (ICIs) that target either programmed death 1 (PD-1) or programmed death ligand 1 (PD-L1) have shown tremendous progress in the treatment of various types of cancer, including the ESCC [6]. The efficacy of ICIs as a second-line therapy for patients with advanced or metastatic ESCC has been verified in several randomized controlled trials (RCTs) [7–11]. Nivolumab (Niv), the first ICI available in China, has demonstrated an overall survival (OS) benefit over chemotherapy (median 10.9 vs. 8.4 months, Hazard ratios (HRs) = 0.77, 95%CI 0.62 to 0.96) [8]. In 2020, the ESCORT trial indicated that camrelizumab (Cam) significantly prolonged OS (median 8.3 vs. 6.2 months, HR = 0.71, 95%CI 0.57 to 0.87) in comparison to chemotherapy for patients with ESCC [7]. The subsequent KEYNOTE-181, ORIENT-2, and RATIONALE-302 studies also revealed that second-line treatment with pembrolizumab (Pem), sintilimab (Sin), or tislelizumab (Tis) significantly improved overall survival in patients with advanced or metastatic ESCC [9–11]. Recently, a meta-analysis showed a statistically significant improvement in objective response rate (*P* = 0.007) and overall survival (*P* = 0.001) with the use of ICIs [12]. As a result, the aforementioned ICIs have been recommended by the Chinese Esophageal Cancer Treatment Guidelines for advanced ESCC patients who have failed first-line treatment [13].

The ICIs were widely used in clinical practice due to their encouraging clinical benefits. Nevertheless, the economic burden on individuals and healthcare systems has increased significantly as new therapeutic technologies provide greater clinical benefits. Notably, health expenditures for esophageal cancer treatment have shown an annual increase of approximately 10% from 2002 to 2011, and the average annual growth rate of medical expenses for Chinese patients with esophageal cancer has exceeded 6% between 2011 and 2015 [14, 15]. Therefore, the cost-effectiveness of ICIs deserves further investigation under the current healthcare policy in China. In this study, an economic model was conducted to evaluate the cost-effectiveness of pembrolizumab, sintilimab, camrelizumab, nivolumab, and tislelizumab as second-line treatments for locally advanced or metastatic ESCC from the perspective of the Chinese healthcare system.

## Materials and methods

### Network meta-analysis

Due to the lack of head-to-head clinical trials, a Bayesian network meta-analysis (NMA) was performed. The network meta-analysis was conducted in accordance with the PRISMA guidelines (Supplementary eTable 3) [16]. A comprehensive search of PubMed, the Cochrane Central Register of Controlled Trials databases, and the Embase database for the past 10 years was conducted using the terms: (pembrolizumab OR nivolumab OR camrelizumab OR sintilimab OR tislelizumab OR PD-1 OR PD-L1) AND (“esophageal squamous cell cancer” OR “esophageal cancer” or “esophageal carcinoma”) AND random*. Two investigators independently identified eligible RCTs that compared ICIs with other regimens in patients with locally advanced or metastatic ESCC who have failed first-line therapy. Studies that were not randomized, duplicated, or in which the drug was unavailable in China were excluded. The risk of bias in clinical trials was assessed using RevMan software (version 5.2) according to the Guides and handbooks of Cochrane [17].

Study characteristics, treatment regimens, and HRs of progression-free survival (PFS) and OS were collected. The pooled HRs were calculated based on the ln(HR) and seln(HR) [18]. Fixed-effects models were employed due to insufficient data to evaluate heterogeneity across trials. Given the lack of a closed loop for indirect comparisons, the consistency test was not performed. We generated four independent Markov chains to estimate the posterior distribution, employing 10,000 adaptation iterations and 20,000 inference iterations per chain. The surface under the cumulative ranking curve (SUCRA) was applied to rank the different regimens. The Bayesian NMA was carried out using the “gemtc” package in R software (version 4.3.1).

### Population and interventions

The cost-effectiveness analysis was conducted and reported following the Comprehensive Health Economic Evaluation Reporting Standards (CHEERS) (Supplementary eTable 4) [19]. The target population for the economic model was in compliance with the eligibility criteria of RCTs. These hypothetical patients had a diagnosis of locally advanced or metastatic ESCC, and either progressed or experienced intolerance to first-line chemotherapy. All enrolled ESCC patients were divided into five groups. Each group received a specific treatment regimen as follows: (1) nivolumab arm, administered intravenously 240 mg every 2 weeks; (2) camrelizumab arm, administered intravenously 200 mg every 2 weeks; (3) sintilimab arm, administered intravenously 200 mg every 3 weeks; (4) pembrolizumab arm, administered intravenously 200 mg every 3 weeks; (5) tislelizumab arm, administered intravenously 200 mg every 3 weeks. Participants in the treatment arm will receive ICIs until confirmed disease progression, intolerable toxicity, or death.

### Model structure

**Fig. 1:**
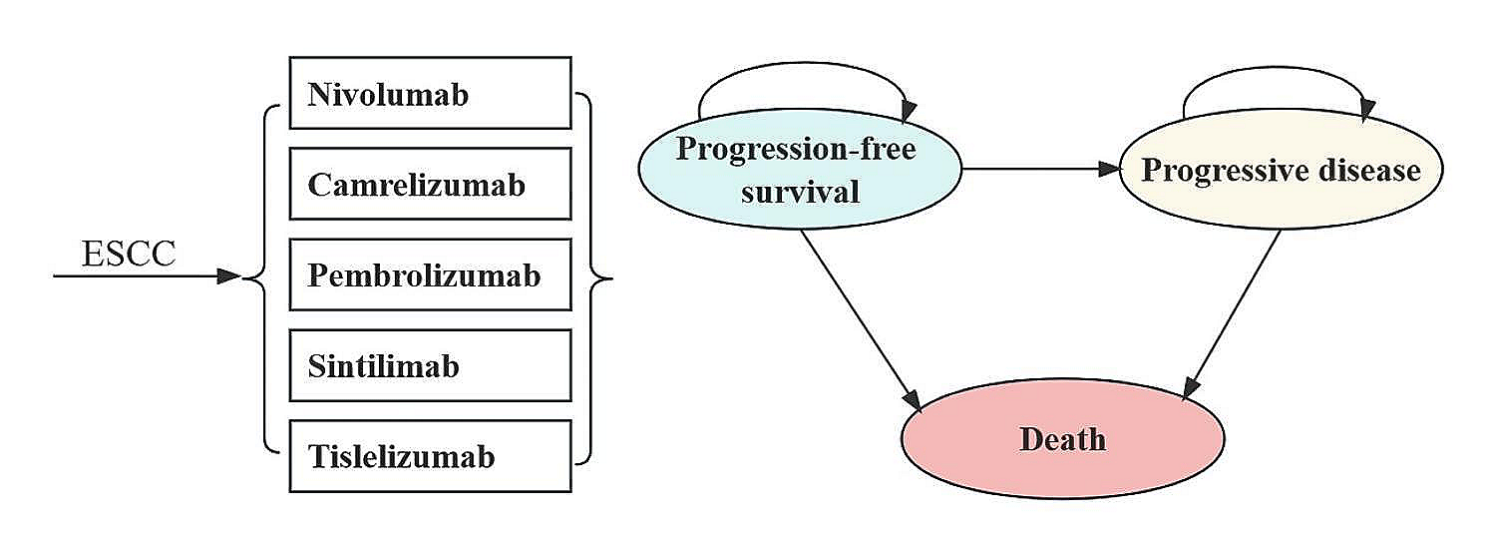
The simplified Markov state transition model. *ESCC, esophageal squamous cell carcinoma*

A Markov model was utilized to simulate the progression of ICIs in the treatment of advanced or metastatic ESCC. Three mutually exclusive health states were incorporated in the economic model: PFS, Progressive Disease (PD), and Death. In accordance with the process of disease progression, it was assumed that the PFS state was the initial state and that death was the absorbing state (Fig. 1). A three-week cycle duration was set for our model. The time horizon for our model was 10 years, during which approximately 99% of patients transitioned to mortality. This model was established using Treeage Pro Suite 2011 software.

The prime evaluation indicator for cost-effectiveness was the incremental cost-effectiveness ratio (ICER), which was calculated from the total cost and quality-adjusted life-years (QALYs). According to the Chinese guidelines for pharmacoeconomic evaluations, an annual discount rate of 5% was used for both cost and health utility from the second year of the model to reflect the net present value [20]. The willingness-to-pay (WTP) threshold was set at three times China’s per capita gross domestic product (GDP) in 2022 ($38,223.34/QALY) to evaluate the cost-effectiveness of the five competing treatment options [20]. All costs were converted to US dollars using the 2022 annual exchange rate (US$1.00 = ¥6.7261).

### Transition probabilities

The transition probabilities between each state of the Markov model were calculated primarily based on clinical efficacy. PFS and OS data for nivolumab at multiple time points were generated from the Kaplan-Meier (KM) curves of the ATTRACTION-3 trial using WebPlotDigitizer (https://apps.automeris.io/wpd/index.zh_CN.html). Individual patient data (IPD) were reconstructed using the “survHE” package in the R software to fit parametric models, such as Weibull, exponential, Gompertz, Log-Logistic and Log-Normal. Akaike Information Criterion (AIC) and Bayesian Information Criterion (BIC) were used to evaluate the best-fitting distributions (Supplementary eFigure 6). As a result, the Log-normal function was selected to simulate both the PFS and OS curves of nivolumab (Supplementary eFigure 7). The fitted distributions for the remaining four treatment regimens were calculated based on the fitted function of nivolumab and the HRs from the above NMA [21].

The transition probability from PFS to PFS was calculated with fitted PFS parametric survival models, as following formulation: Prob_(PFS−PFS)_ = S(t)/S(t-1), where t presents the current cycle of the Markov model. The annual natural mortality rate (7.37‰) of China in 2022 [22] was applied to calculate the transition probability from PFS to death. The overall mortality rate at each cycle was calculated using the fitted OS parametric survival models. Subsequently, transitioning probability from PD to PD was calculated with natural and overall mortality rates. The transition probability from PFS to PD is equal to 1 minus the transition probability from PFS to PFS and the transition probability from PFS to death. The transition probability from PD to death is equal to 1 minus the transition probability from PD to PD.

### Cost and utility

Based on the perspective of Chinese healthcare, only direct medical expenditures were included in the model, including anticancer agents, hospitalization, follow-up, and adverse events (AEs) management (Table 1). Hospitalization costs include the cost of beds, nursing care, and the dispensing and injection of anticancer drugs. According to Guidelines for the Management of ICI-related Toxicity by the Chinese Society of Clinical Oncology (CSCO) [23] and clinical practice, computed tomography (CT), electrocardiogram, ultrasonography, and laboratory tests were considered in the follow-up of PFS state. The laboratory tests included routine blood, routine urine, routine stool, biochemical, thyroid, coagulation, etc. CT scans were assumed to be performed once every six weeks, while other examinations were performed before each dose. Expenditures for PD status consisted of the cost of subsequent systemic anticancer therapies (SSAT), follow-up, best supportive care (BSC), and end-of-life care. Subsequent therapy was not available to all treatment groups. Therefore, it was assumed that subsequent treatment after disease progression was consistent across all five intervention groups. The model applied subsequent treatment after the progression of nivolumab from ATTRACTION-3, and only regimens with greater than 1% patient use were included [8]. Given that the proportion of male patients in all five RCTs was above 84%, we calculated the subsequent chemotherapy dosage using the average weight (69.6 kg) and height (169.7 cm) of Chinese males to simplify the calculation [24]. Only the AEs (grade ≥ 3) with incidence rates greater than 1% were included in the analysis. The costs associated with AEs management were gathered from published literature [21, 25–27] and were only used once during the initial cycle of the model. All costs were sourced from local charges or previously published studies. We adjusted the costs of literary resources using the consumer price index (CPI), if necessary.

**Table 1:** Model parameters and assumptions

Parameters	Value	Range		Distribution	Reference
		Lower	Upper		
**Clinical Date**					
Log-normal PFS model of nivolumab	µ = 1.3480; σ = 1.0687				[8]
Log-normal OS model of nivolumab	µ = 2.6273; σ = 1.1419				[8]
HRs of regimens	Network Meta-analysis (eTable 2)			Log-normal	
**Costs (US$)**					
Camrelizumab 200 mg	383.08	306.46	459.70	Gamma	Local charge
Sintilimab 100 mg	160.57	128.46	192.68	Gamma	Local charge
Nivolumab 240 mg	3,436.02	2,748.82	4,123.22	Gamma	Local charge
Tislelizumab 100 mg	204.80	163.84	245.76	Gamma	Local charge
Pembrolizumab 100 mg	2,663.95	2,131.16	3,196.74	Gamma	Local charge
CT/Unit	50.10	40.08	60.12	Gamma	[28]
ECG/Unit	2.97	2.38	3.56	Gamma	[28]
Ultrasonography/Unit	11.15	8.92	13.38	Gamma	[28]
Laboratory tests of PFS/Unit	206.06	164.85	247.27	Gamma	[28]
Laboratory tests of PD/Unit	237.43	189.94	284.92	Gamma	[28]
Drug dispensing/Unit	2.51	2.01	3.01	Gamma	[28]
Intravenous injection/Unit	2.78	2.22	3.34	Gamma	[28]
Nursing fees/Day	1.49	1.19	1.79	Gamma	[28]
Bed fees/Day	5.95	4.76	7.14	Gamma	[28]
Docetaxel/20 mg	4.46	3.57	5.35	Gamma	Local charge
Paclitaxel/100 mg	27.58	22.06	33.10	Gamma	Local charge
Cisplatin/30 mg	2.84	2.27	3.41	Gamma	Local charge
Nedaplatin/10 mg	7.72	6.18	9.26	Gamma	Local charge
Fluorouracil/250 mg	4.37	3.50	5.24	Gamma	Local charge
TGO/42 capsules	33.78	27.02	40.54	Gamma	Local charge
BSC/Cycle	118.46	94.77	142.16	Gamma	[25]
End-of-life care	1,489.51	1,191.60	1,787.41	Gamma	[25]
**Cost of AEs (US$)**					
Asthenia	109.14	87.31	130.97	Gamma	[25]
Diarrhea	50.18	40.15	60.22	Gamma	[26]
Hyponatraemia	3223.00	2578.40	3867.60	Gamma	[27]
Anemia	143.21	114.57	171.85	Gamma	[25]
Pulmonary inflammation	1,017.36	813.89	1,220.83	Gamma	[26]
Neutropenia	118.70	94.96	142.44	Gamma	[25]
Thrombocytopenia	1,554.30	1,243.44	1,865.16	Gamma	[25]
Lymphopenia	118.70	94.96	142.44	Gamma	[25]
Lung infection	1672.80	1,338.24	2,007.36	Gamma	[25]
AST increased	71.06	56.85	85.27	Gamma	[21]
ALT increased	71.06	56.85	85.27	Gamma	[21]
GGT increased	71.06	56.85	85.27	Gamma	[21]
**Probabilities of AEs in Nivolumab Arm (%)**					
Anemia	1.9	1.5	2.3	Beta	[8]
Probabilities of AEs in Camrelizumab Arm (%)					
Asthenia	1.3	1.0	1.6	Beta	[7]
Anemia	2.6	2.0	3.1	Beta	[7]
Diarrhea	1.3	1.0	1.6	Beta	[7]
Hyponatraemia	1.3	1.0	1.6	Beta	[7]
**Probabilities of AEs in Pembrolizumab Arm**					
Asthenia	1.3	1.0	1.6	Beta	[9]
Anemia	1.3	1.0	1.6	Beta	[9]
**Probabilities of AEs in Sintilimab Arm**					
Pulmonary inflammation	5.3	4.2	6.4	Beta	[11]
Neutropenia	2.1	1.7	2.5	Beta	[11]
Thrombocytopenia	2.1	1.7	2.5	Beta	[11]
Lymphopenia	2.1	1.7	2.5	Beta	[11]
Lung infection	2.1	1.7	2.5	Beta	[11]
**Probabilities of AEs in Tislelizumab Arm**					
Anemia	3.0	2.4	3.6	Beta	[10]
AST increased	2.7	2.2	3.2	Beta	[10]
ALT increased	1.3	1.0	1.6	Beta	[10]
GGT increased	4.0	3.2	4.8	Beta	[10]
Neutropenia	1.7	1.4	2.0	Beta	[10]
**Probabilities of Subsequent systemic anticancer therapies (%)**					
Docetaxel	21.0	16.8	25.2	Beta	[8]
Paclitaxel	35.7	28.6	42.8	Beta	[8]
Cisplatin	6.7	5.4	8.0	Beta	[8]
Nedaplatin	1.9	1.52	2.3	Beta	[8]
Fluorouracil	5.7	4.6	6.8	Beta	[8]
TGO	6.2	5.0	7.4	Beta	[8]
**Utilities**					
PFS	0.74	0.59	0.89	Beta	[29]
PD	0.58	0.46	0.70	Beta	[29]
**Disutilities of AEs**					
Asthenia	0.07	0.06	0.08	Beta	[25]
Diarrhea	0.07	0.06	0.08	Beta	[30]
Hyponatraemia	0.03	0.02	0.04	Beta	[27]
Anemia	0.07	0.06	0.08	Beta	[25]
Pulmonary inflammation	0.05	0.04	0.06	Beta	[25]
Neutropenia	0.20	0.16	0.24	Beta	[25]
Thrombocytopenia	0.11	0.09	0.13	Beta	[25]
Lymphopenia	0.20	0.16	0.24	Beta	[25]
Lung infection	0.05	0.04	0.06	Beta	[25]
AST increased	0.12	0.10	0.14	Beta	[21]
ALT increased	0.12	0.10	0.14	Beta	[21]
GGT increased	0.12	0.10	0.14	Beta	[21]
**Others**					
Discount rate (%)	5	0	8	Beta	[20]
BSA (m2)	1.77	1.42	2.12	Gamma	[24]

AEs, Adverse events; ALT, alanine aminotransferase; AST, aspartate aminotransferase; BSA, body surface area; BSC, best supportive care; CT, Computed Tomography; ECG, Electrocardiogram; PFS, progression-free survival; GGT, γ-glutamyl transpeptidase; HRs, hazard ratios; OS, overall survival; TGO, Tegafur/Gimeracil/Oteracil Potassium

Health utility is a quantification of the health-related quality of life for each health state. Utility values for each health state and disutility values for AEs were derived from previously published studies [21, 25, 27, 29, 30]. Utility values were assumed to be equal across treatment groups for the same health state. The disutility of adverse events was only considered in the initial cycle of the model, similar to the AEs management costs.

### Sensitivity analysis

Univariate sensitivity analysis and probabilistic sensitivity analysis (PSA) were conducted to evaluate the uncertainty of the model. In the univariate sensitivity analysis, parameter variables were allowed to vary within a specific range of 95% confidence intervals or ± 20% deviation from baseline values (Table 1). In addition, the discount rate varied between 0 and 8%. The results of univariate sensitivity analysis were presented in tornado diagrams. In the PSA, a Monte Carlo simulation of 1,000 iterations was conducted to evaluate the impact of model variables that follow particular distributions. Log-normal distribution was utilized for HRs between competing regimens, while gamma distribution was employed for costs. Additionally, beta distribution was used for proportions, incidence rates, and utility values (Table 1). A scatter plot of 1000 iterations and the cost-effectiveness acceptability curve were used to display the results of the PSA.

Furthermore, given the preliminary results of the base-case, scenario analyses were conducted to explore the price reductions required for camrelizumab and tislelizumab to be cost-effective versus sintilimab.

## Results

### Network meta-analysis

A total of 352 records were identified using the search strategy, and five clinical trials with 1,797 patients were eventually included in the network meta-analysis (Supplementary eFigure 1). The basic characteristics of the included studies were shown in Supplementary eTable 1. Meanwhile, Supplementary eFigure 2 provided the potential bias risk for each included research. It can be seen that the RCTs included in the analysis exhibited bias primarily in regard to blinding. The network plot of the analysis was displayed in the Supplementary eFigure 3. The trace and density plots were shown in the Supplementary eFigure 4. The SUCRA result indicated that camrelizumab and tislelizumab achieved the highest ranks for both progression-free survival and overall survival (Supplementary eFigure 5). The results of pooled HRs were reported in Supplementary eTable 2. It revealed that camrelizumab exhibited a significantly longer PFS compared to nivolumab (HR 0.64; 95% CI 0.47–0.87). Additionally, no other significant differences in PFS or overall OS were noted when comparing the other ICIs in our study.

### Base-case of cost-effectiveness analysis

Over a 10-year horizon, the sintilimab-treated group was expected to cost the least, and the pembrolizumab-treated group was expected to cost the most. Camrelizumab provided the greatest clinical benefit closely followed by tislelizumab, which was similar to the SUCRA ranking. Compared to sintilimab, the costs of tislelizumab and camrelizumab were $1,392.27 and $4,641.74 more, respectively. This resulted in ICERs of $75,472.65/QALY, and $175,681.92/QALY. The ICERs for nivolumab and pembrolizumab were negative, driven by higher cost and lower efficacy, suggesting that they were both dominated compared to sintilimab. It revealed that sintilimab could be considered the most cost-effective option based on the WTP threshold of $38,223.34/QALY. The base-case results were summarized in Table 2.

**Table 2:** Results of the base-case analysis

Regimen	Cost ($)	QALYs	ICER ($/QALY)				
Sintilimab	9,662.47	0.953	Sintilimab				
Tislelizumab	11,054.76	0.971	75,472.65	Tislelizumab			
Camrelizumab	15,696.50	0.987	175,681.92	291,956.23	Camrelizumab		
Nivolumab	44,120.77	0.861	Dominated	Dominated	Dominated	Nivolumab	
Pembrolizumab	52,885.34	0.867	Dominated	Dominated	Dominated	1,454,223.31	Pembrolizumab

ICER, incremental cost-effectiveness ratio; QALY, quality-adjusted life year

### Sensitivity analysis

**Fig. 2:**
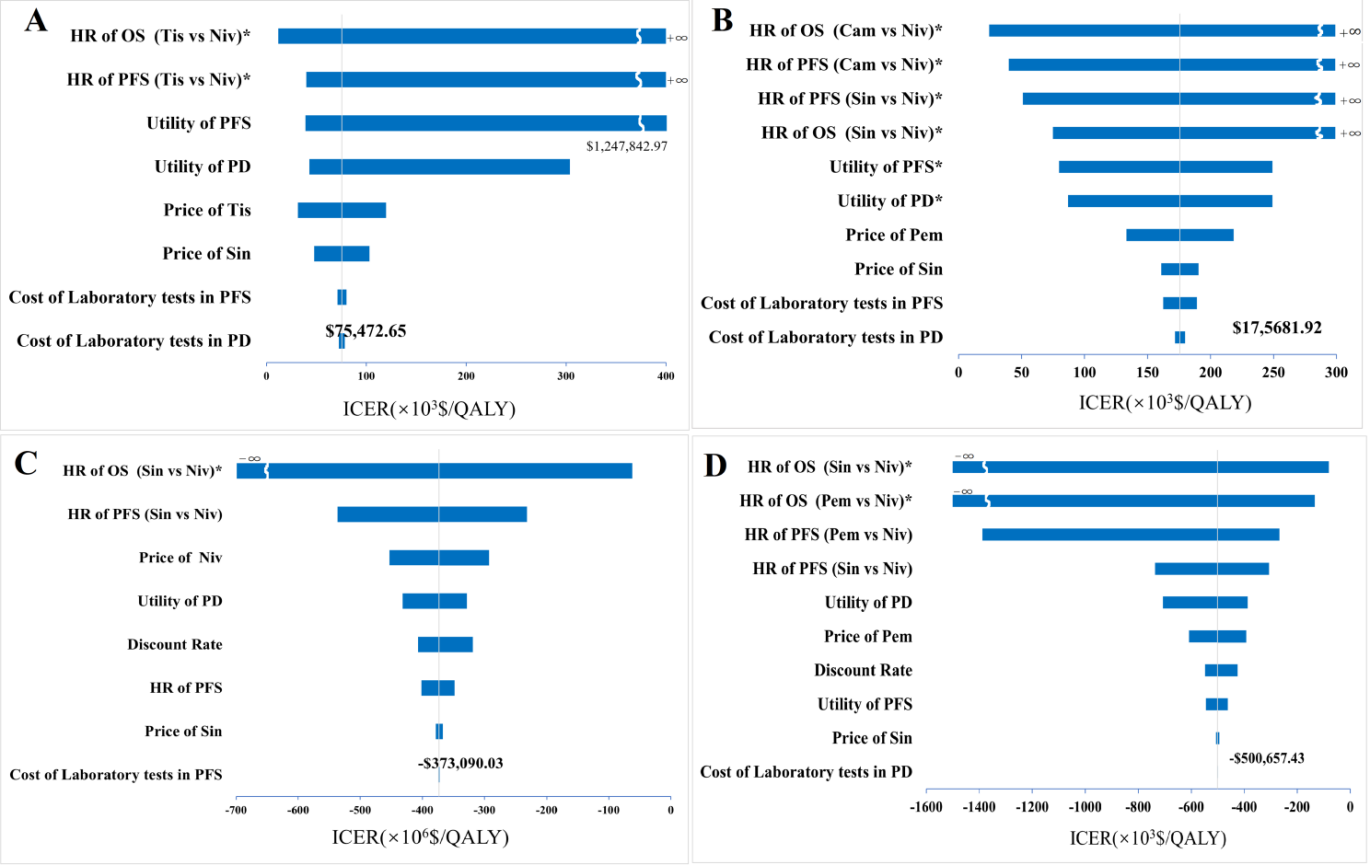
Tornado diagrams of univariate sensitivity analysis. **(A)** Tislelizumab vs. Sintilimab; **(B)** Camrelizumab vs. Sintilimab; **(C)** Nivolumab vs. Sintilimab; **(D)** Pembrolizumab VS Sintilimab. PFS, progression-free survival; HRs, hazard ratios; PD, progressive diease; OS, overall survival; Cam, Camrelizumab; Niv, Nivolumab; Pem, Pembrolizumab; Sin, Sintilimab; Tis, Tislelizumab. * QALY appeared the same for both treatment groups during the parameter change, which corresponds to an ICER of positive or negative infinity

Given that the base-case results suggested sunitinib to be the most cost-effective, the sensitivity analyses primarily presented the results of other ICIs in pairwise comparisons with sunitinib. The tornado diagram of the univariate sensitivity analysis result (Fig. 2) showed that the HR_(Tis vs. Niv)_ of OS and PFS, the utilities of PFS and PD, and the price of tislelizumab had the greatest impact on the ICER when tislelizumab was compared to sintilimab. When the HR_(Tis vs. Niv)_ of OS reached to the lower limit (equivalent to an expansion of the clinical effect of tislelizumab), the ICER decreased to $11,864.62, which was below the WTP threshold. If the lower price of tislelizumab was applied, the ICER would fall below the WTP threshold. Comparing camrelizumab to sintilimab, the ICER was substantially sensitive to HRs and the utilities of states. Similarly, sintilimab was no longer cost-effective compared to camrelizumab when the HR_(Cam vs. Niv)_ of OS was at the minimum. For nivolumab versus sintilimab, and pembrolizumab versus sintilimab, HRs were the most sensitive parameters. Overall, all other scenarios did not change the cost-effectiveness conclusion, except for the three cases mentioned above.

**Fig. 3:**
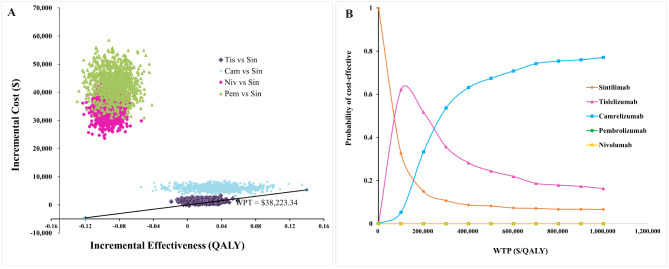
Results of Probability sensitivity analysis. **(A)** Scatter plots of 1000 Monte Carlo simulated patients; **(B)** The cost-effectiveness acceptability curve. Cam, Camrelizumab; Niv, Nivolumab; Pem, Pembrolizumab; Sin, Sintilimab; Tis, Tislelizumab; WTP, Willingness to Pay; QALY, Quality Adjusted Life Year

In the PSA, the probability of tislelizumab, camrelizumab, nivolumab, and pembrolizumab being the most cost-effective option compared to sintilimab was 15.6%, 0%, 0%, and 0%, respectively (Fig. 3B). The cost-effectiveness acceptability curves showed that the probability of sintilimab being cost-effective in simultaneous comparisons of competing regimens was 84.4% at a WTP threshold of $38,223.34/QALY. Tislelizumab was the most cost-effective regimen when the WTP threshold was between $72,133/QALY and $241,000/QALY. When the WTP threshold was greater than $241,000/QALY, camrelizumab was likely the most cost-effective option among the five competing regimens.

The scenario analysis results suggested that sintilimab would always be economically favorable until the price of camrelizumab fell below 35.4% of the current levels, or the price of tislelizumab fell below 83.1% of the current levels.

## Discussion

The expression of PD-1 ligands and the activation of the PD-1 pathway through the binding of appropriate effector cells to the PD-1 receptor are the primary mechanisms by which tumor cells evade the immune response [31]. PD-L1 molecules combat malignant cells by binding to the PD-1 receptor, inducing PD-1 signaling, and suppressing the immune response of T cells. ICIs, such as PD-1/PDL1 inhibitors, have significantly improved survival and quality of life for ESCC patients in either first-line or second-line therapy [12, 27]. However, the high price of ICIs limited their utilization, especially in developing countries. Therefore, objective economic evaluation is necessary for clinical decision-making. We performed a cost-effectiveness analysis to assess the economic outcomes of five ICIs as second-line therapy for patients with locally advanced or metastatic ESCC from the perspective of the Chinese healthcare system. The result of our study revealed that sintilimab was the most cost-effective regimen among the five alternative ICIs at the WTP threshold of 3 times GDP per capita. The PSA results suggested sintilimab had an 84.4% chance of being cost effective. Regardless of changing the WTP threshold, the probabilities of nivolumab and pembrolizumab being cost-effective were consistently zero.

Owing to the absence of direct comparative clinical trials, we performed a network meta-analysis to obtain HRs for indirect comparisons. Five RCTs were included in the network meta-analysis of this study. The baseline characteristics of the patients, such as age, gender, and ECOG score, were similar across the trials, indicating the comparability of the trial populations. The definitions of efficacy and safety outcomes were also consistent. Although the chemotherapy control regimens were slightly different, they all had similarities: tislelizumab and pembrolizumab were compared with paclitaxel, docetaxel, or irinotecan; sintilimab was compared with paclitaxel or irinotecan; and camrelizumab was compared with docetaxel or irinotecan; Nivolumab was compared with paclitaxel or docetaxel. Therefore, this network meta-analysis has high feasibility, and its results can provide an important reference for clinical decision-making. According to the result of the NMA, camrelizumab and tislelizumab had superior SUCRA scores for the second-line treatment of ESCC, which resulted in both of them receiving more QALYs in our cost-effectiveness analysis. Otherwise, the included studies showed that all the median PFS were within 2 months, while the median OS ranged from 7.2 to 10.9 months [7–11]. In the univariate sensitivity analysis, all pairwise comparisons were highly sensitive to the HRs of OS, which may be attributed to the patient experiencing a longer period in the progressive state than in PFS allowing for a greater proportion of QALYs to be gained in the PD state.

In recent years, China’s self-developed innovative ICIs have made excellent progress, and so far more than 10 ICIs have been approved by the National Medical Products Administration (NMA) of China. Three of the five therapeutic regimens in our study were developed by Chinese pharmaceutical companies: sintilimab, tislelizumab, and camrelizumab. There are five approved indications for sintilimab, eight for tislelizumab, and nine for camrelizumab in China. The NMA results suggested that these treatments were as effective as nivolumab and pembrolizumab. Our cost-effectiveness analysis demonstrated that they were superior alternatives compared to nivolumab and pembrolizumab.

Several published studies assessed the cost-effectiveness of ICIs as second-line treatment for ESCC. A partitioned survival model was established by Cai et al. to compare the cost-effectiveness between camrelizumab and chemotherapy based on the perspective of Chinese society [32]. The estimated ICER was $3,999/QALY, which was remarkably lower than the WTP threshold in that study. Shi et al. conducted an economic evaluation comparing tislelizumab to chemotherapy from the perspective of Chinese healthcare payers, and the results indicated that tislelizumab was a likely cost-effective alternative option [29]. However, cost-effectiveness analyses of nivolumab and pembrolizumab, based on the ATTRACTION-3 and KEYNOTE-181 studies, demonstrated that neither of them was cost-effective when compared to chemotherapy in China [33, 34]. Nevertheless, the above pharmacoeconomic studies were designed with chemotherapy as the control group, and there were almost no published studies on cost-effectiveness analyses between different ICIs for second-line treatment of ESCC. To the best of our knowledge, this is the first study to evaluate the cost-effectiveness of the five alternative ICIs for treating ESCC in the second line. The results proved that sintilimab was more likely to be cost-effective.

China is a developing country, and most patients have limited ability to afford expensive treatment regimens. It was not uncommon for patients to become poor due to illness, especially in rural areas. Hence, obtaining the maximum health outcome with limited medical resources is a difficult problem for patients and doctors to face inevitably. After the establishment of the National Healthcare Security Administration (NHSA) in 2018, national medical insurance system reform has made great strides forward in China. Through a process of price negotiation, expensive innovative agents were admitted to the National Reimbursement Drug List (NRDL), enabling them eligible for Medicare reimbursement. Sintilimab, tislelizumab, and camrelizumab were added to the NRDL in 2021, with corresponding price reductions of approximately 64%, 80%, and 85%. The National Drug Price Negotiation Project has improved the accessibility and affordability of ICIs in China. Nivolumab and pembrolizumab were not listed on the NRDL, and they exhibited substantially higher per-cycle drug costs compared to the other three agents.

In the latest NRDL, tislelizumab, and camrelizumab are eligible for reimbursement as second-line therapy in cases of locally advanced or metastatic ESCC. However, sintilimab is not eligible for reimbursement in this indication [35]. Our analysis indicated that the sintilimab was likely the most cost-effective among the five competing ICIs, which may provide economic evidence for updating the NRDL in the future. In addition, according to our scenario analysis, camrelizumab would be more cost-effective than sintilimab if its price were reduced by 64.6%. Similarly, tislelizumab would also be cost-effective compared to sintilimab if its price were reduced by 16.9%. This may also be a factor to consider when updating the NRDL.

There are several limitations in our study. First, due to the lack of head-to-head clinical trials among the five alternative ICIs, the NMA was employed for indirect comparison in our study. It is important to note that any biases present in these clinical trials may affect the results of our study. Furthermore, the ATTRACTION-3, KEYNOTE-181, and RATIONALE-302 trials enrolled non-Asian populations, potentially introducing a bias when applied to cost-effectiveness analyses tailored to the Chinese healthcare system. Additionally, our cost-effectiveness model revealed a relatively small variance in QALY gains for ESCC patients across different ICI regimens. This minimal discrepancy aligns with the NMA findings, which indicate limited overall differences in clinical efficacy between the ICIs. This limitation cannot be avoided currently, incorporating more clinical studies into NMA in the future may improve the accuracy of economic outcomes. Second, it was assumed that all five intervention groups would receive identical treatment after disease progression. However, in reality, the treatment regimens will vary depending on the patient’s condition. Third, only serious adverse events were considered in the model. Mild adverse events were excluded from our analysis as they were assumed to be self-limiting. However, the univariate sensitivity analyses showed that varying AE-related parameters had a small impact on results. Fourth, our model did not account for the potential heterogeneity of the patient population and corresponding subgroup analyses. The analysis did not consider the expression of biomarkers that had a notable influence on both clinical efficacy and cost-effectiveness. Finally, the sensitivity analyses assumed a uniformly 20% range of variation above and below the baseline value for variables where the standard error or confidence intervals were not reported. Although this is a common approach for economic assessments, it may not be suitable for all variables. Despite the limitations, we believe that this study provides an accurate reflection of the economics of ICIs as a second-line therapy for esophageal cancer in China.

## Conclusion

In summary, sintilimab was the most cost-effective option for second-line treatment of patients with advanced or metastatic ESCC compared to the other competing ICIs in China. A 64.6% reduction in the price of camrelizumab would make camrelizumab more cost-effective than sintilimab; a 16.9% reduction in the price of tislelizumab would make tislelizumab more cost-effective than sintilimab. Nivolumab and pembrolizumab were dominated due to higher costs but fewer QALYs gained.

### Electronic supplementary material

Below is the link to the electronic supplementary material.

Supplementary Material 1

## Data Availability

The datasets used and/or analysed during the current study available from the corresponding author on reasonable request.
