# Supplementary material for: Immune checkpoint inhibitors as the second-line treatment for advanced esophageal squamous cell carcinoma: a cost-effectiveness analysis based on network meta-analysis
Source: BMC Cancer. 2024 May 29;24:654. doi: 10.1186/s12885-024-12423-2 (PMC11134960; doi:10.1186/s12885-024-12423-2)
Supplement: Supplementary file 1 — Supplementary Material 1 [file 12885_2024_12423_MOESM1_ESM.docx]

Supplementary Tables and Figures

eFigure 1. Flow diagram of selection process for included studies.

eFigure 2. Risk of bias assessment results.

eFigure 3. Network plot of the network meta-analysis.

eFigure 4. The trace and density plots for the network meta-analysis. (A) progression-free survival; (B) Overall survival.

eFigure 5. SUCRA score of each regimen in the network meta-analysis.

eFigure 6. AIC and BIC of parametric models.

eFigure 7. Model-fitted versus original KM curves for Nivolumab.

eTable 1. Characteristics of the Studies included in the network meta-analysis.

eTable 2. Results of network meta‐analysis for PFS and OS.

eTable 3. PRISMA 2020 Checklist

eTable 4. CHEERS 2022 Checklist


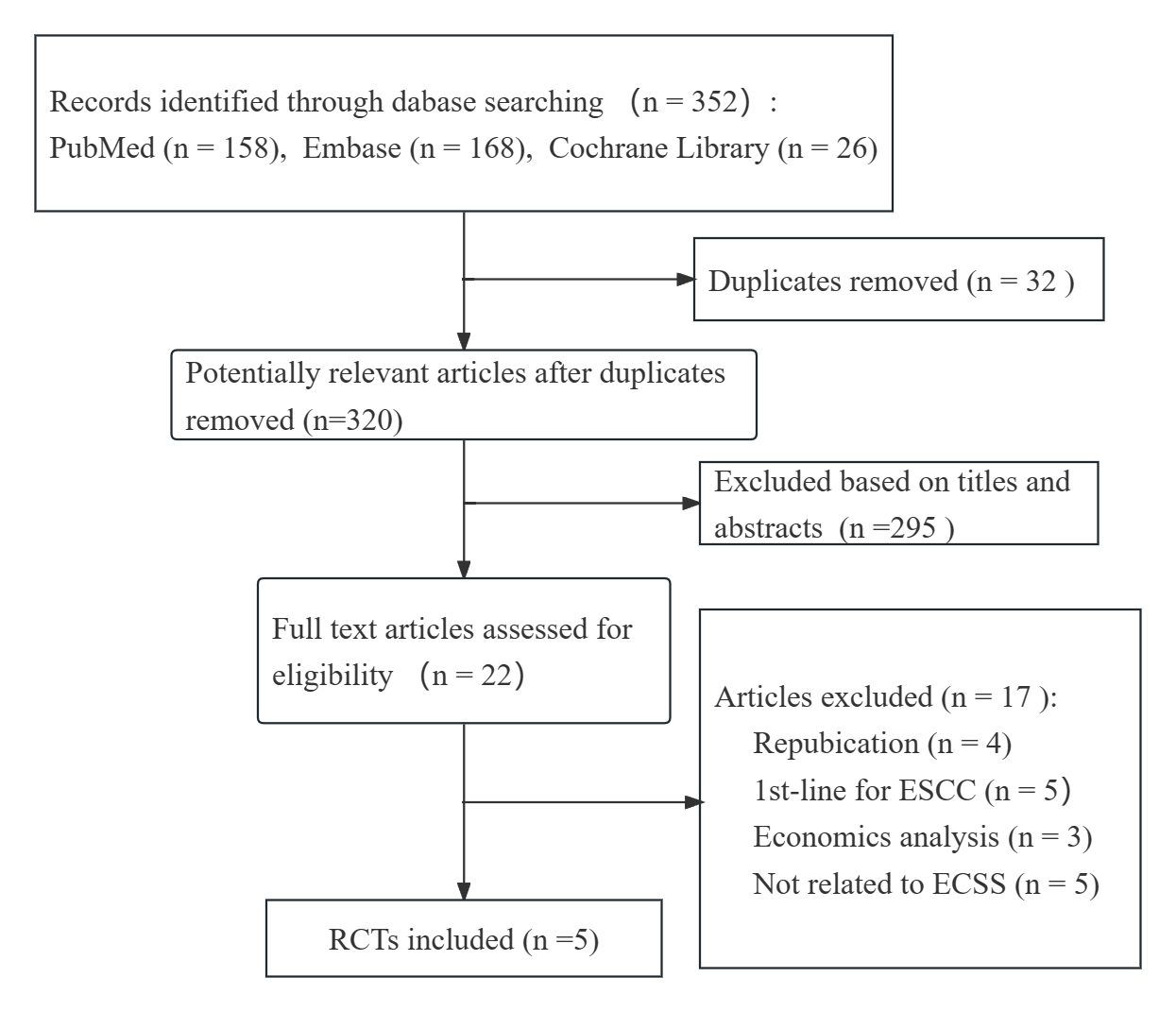


**eFigure 1. Flow diagram of selection process for included studies.**


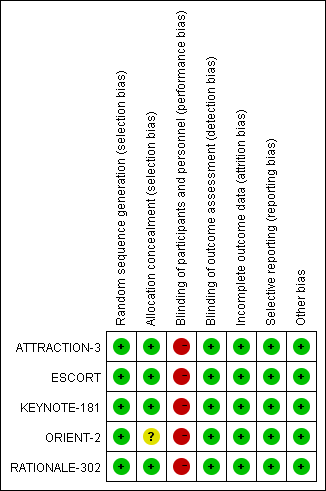


**eFigure 2. Risk of bias assessment results.**


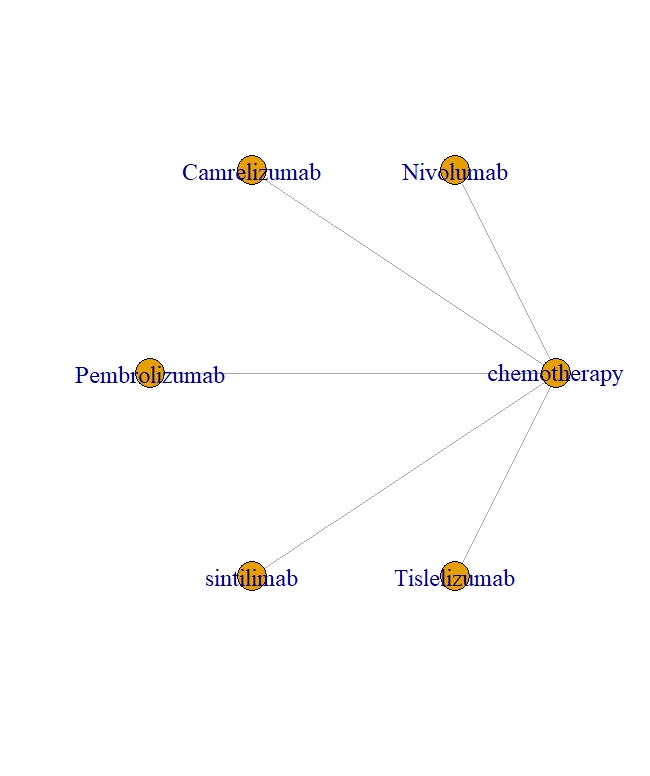


**eFigure 3. Network plot of the network meta-analysis**


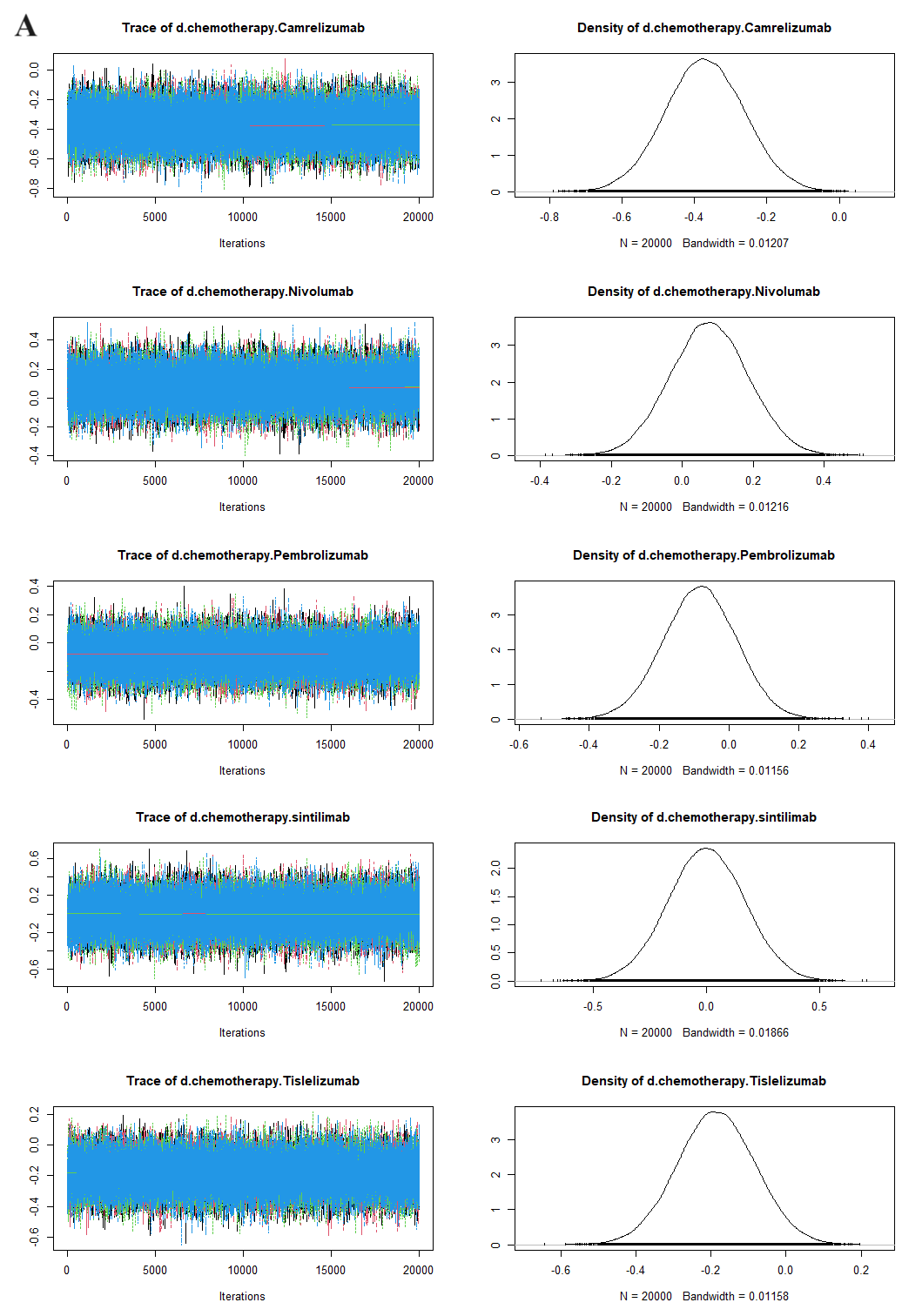

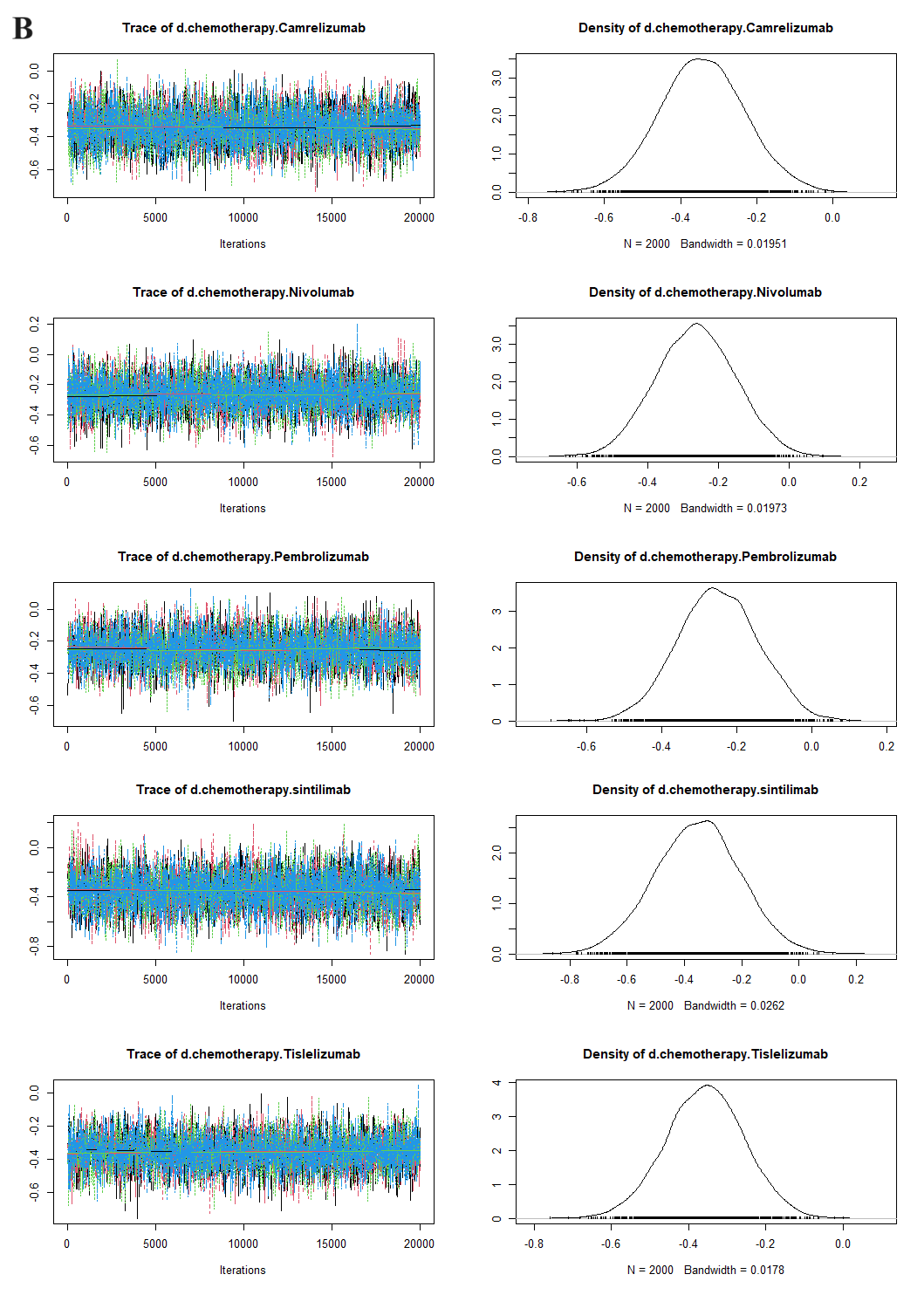


eFigure 4. The trace and density plots for the network meta-analysis. (A) progression-free survival; (B) overall survival.


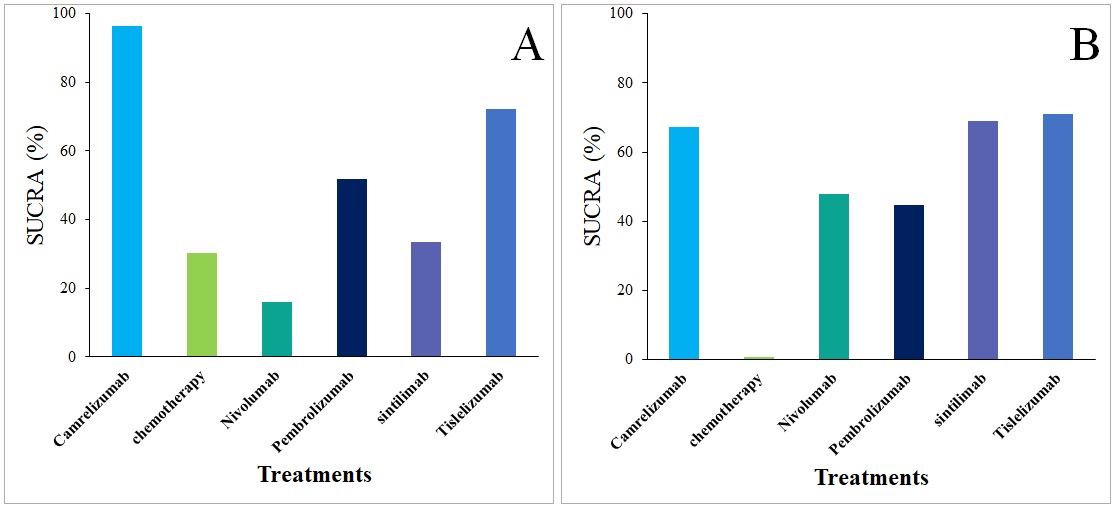


**eFigure 5. SUCRA score of each regimen in the network meta-analysis. (A) progression-free survival; (B) Overall survival.** *SUCRA, surface under the cumulative ranking curve.*


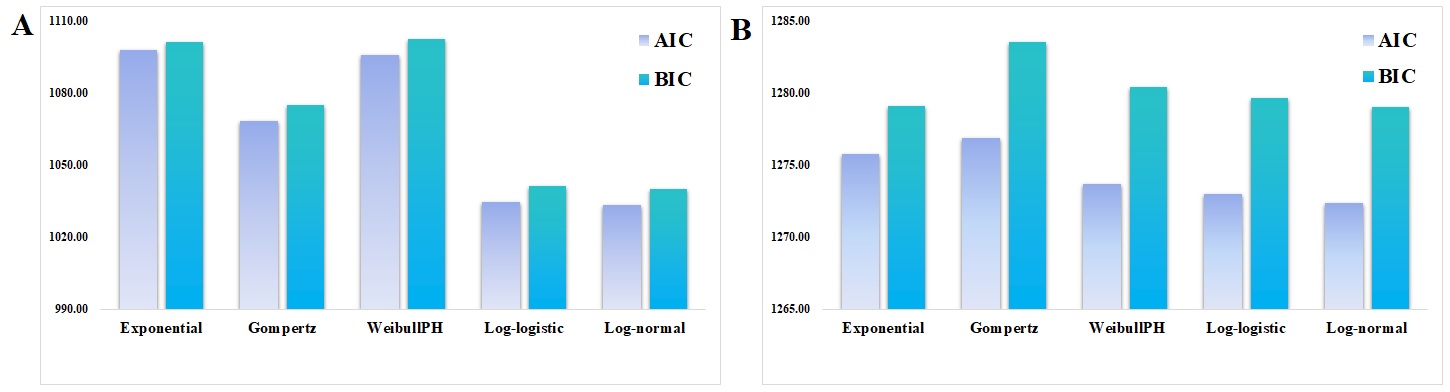


**eFigure 6. AIC and BIC of parametric models. (A) progression-free survival; (B) Overall Survival.** *AIC, Akaike information criterion; BIC, Schwarz Bayesian criterion.*


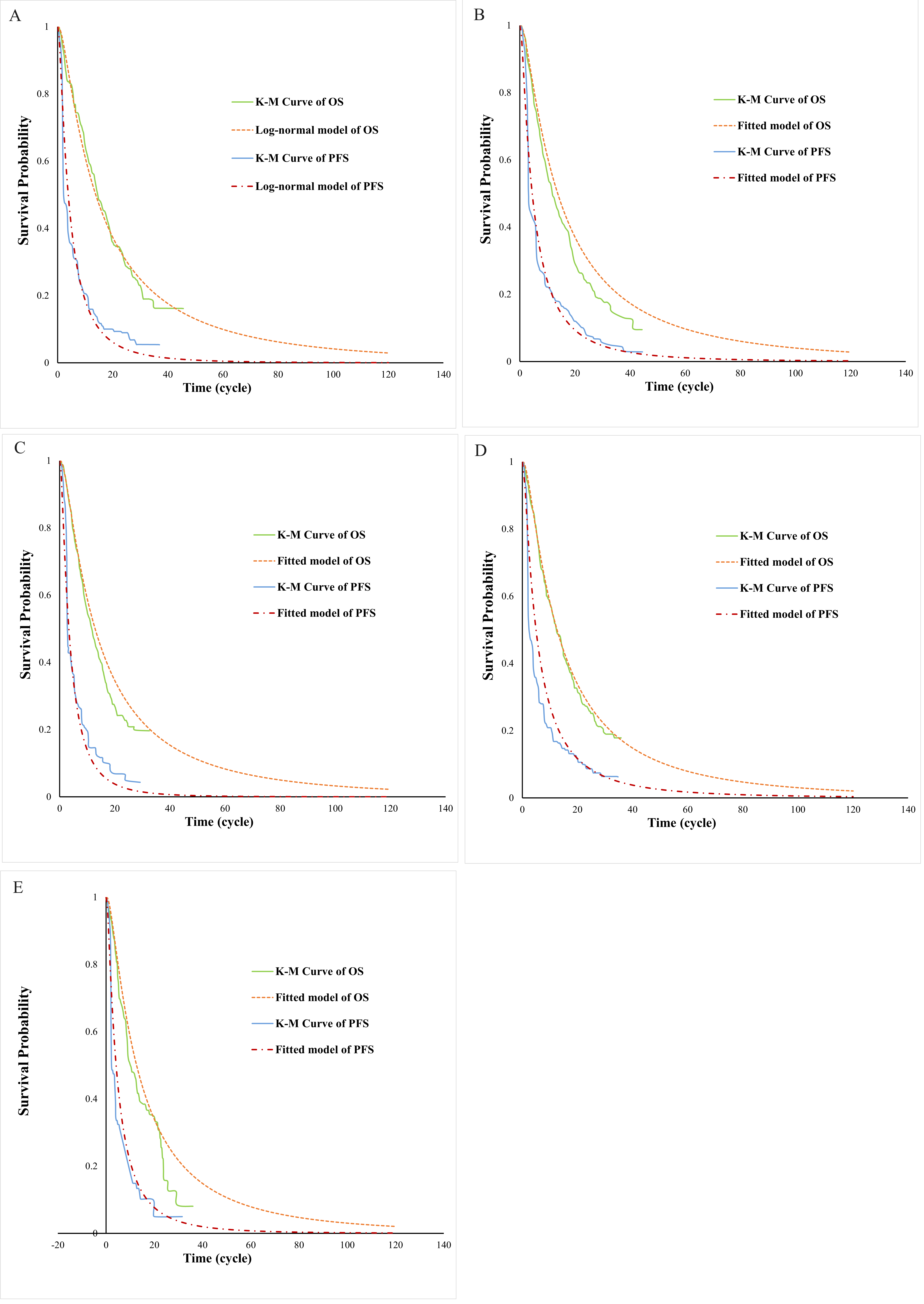


**eFigure 7. Model-fitted versus original KM curves. (A) Nivolumab, (B) Pembrolizumab, (C) Camrelizumab, (D) Tislelizumab, (E) Sintilimab.**

*PFS, progression-free survival; OS, overall survival; KM, Kaplan Meier.*

**eTable 1. Characteristics of the Studies included in the network meta-analysis**

| Study | Author | Year | Phase | Sample size | Male (%) | Age, Years  Median (IQR or Range) | | Intervention | Control | HR of PFS (95% CI) | HR of OS (95% CI) |
| --- | --- | --- | --- | --- | --- | --- | --- | --- | --- | --- | --- |
|  |  |  |  |  |  | Intervention | Control |  |  |  |  |
| ATTRACTION-3 | Kato. et al | 2019 | Ⅲ | 419 | 364 (86.9) | 64 (57–69) | 67 (57–72) | Nivolumab | Chemotherapy | 1.08 | 0.77 |
|  |  |  |  |  |  |  |  |  |  | (0.87-1.34) | (0.62-0.96) |
| ESCORT | Jing. et al | 2020 | Ⅲ | 448 | 400 (89.3) | 60 (54–65) | 60 (54–65) | Camrelizumab | Chemotherapy | 0.69 | 0.71 |
|  |  |  |  |  |  |  |  |  |  | (0.56-0.86) | (0.57-0.88) |
| KEYNOTE-181 | Kojima. et al | 2020 | Ⅲ | 401 | 337 (84.0) | 63.0 (23-84)^*^ | 62.0 (24-84)^*^ | Pembrolizumab | Chemotherapy | 0.92 | 0.78 |
|  |  |  |  |  |  |  |  |  |  | (0.75-1.13) | (0.63-0.96) |
| ORIENT-2 | Xu. et al | 2022 | Ⅱ | 190 | 172 (90.5) | 60 (54–64 | 60 (54–64) | sintilimab | Chemotherapy | 1.00 | 0.70 |
|  |  |  |  |  |  |  |  |  |  | (0.72-1.39) | (0.50-0.97) |
| RATIONALE-302 | Shen. et al | 2022 | Ⅲ | 512 | 432 (84.4) | 62.0 (40-86) | 63.0 (35-81) | Tislelizumab | Chemotherapy | 0.83 | 0.70 |
|  |  |  |  |  |  |  |  |  |  | (0.67-1.01) | (0.57-0.85) |

**Median age of all included patients.*

*HR, hazard ratio; PFS, progression-free survival; OS, overall survival; CL, Confidence interval; IQR, interquartile range.*

**eTable 2. Results of network meta‐analysis for PFS and OS (HR, 95% CL)**

| **PFS** | | | | | |
| --- | --- | --- | --- | --- | --- |
| chemotherapy | 1.08 (0.87, 1.34) | 0.69 (0.56, 0.86) | 0.92 (0.75, 1.13) | 1.00 (0.72, 1.39) | 0.83 (0.68, 1.02) |
| 0.93 (0.75, 1.15) | Nivolumab | 0.64 (0.47, 0.87) | 0.85 (0.63, 1.14) | 0.92 (0.62, 1.37) | 0.77 (0.57, 1.04) |
| 1.45 (1.17, 1.80) | 1.57 (1.15, 2.12) | Camrelizumab | 1.33 (0.99, 1.79) | 1.45 (0.98, 2.15) | 1.20 (0.89, 1.62) |
| 1.09 (0.89, 1.33) | 1.17 (0.87, 1.58) | 0.75 (0.56, 1.01) | Pembrolizumab | 1.09 (0.74, 1.59) | 0.90 (0.68, 1.2) |
| 1.00 (0.72, 1.39) | 1.08 (0.73, 1.60) | 0.69 (0.47, 1.02) | 0.92 (0.63, 1.35) | sintilimab | 0.83 (0.57, 1.22) |
| 1.21 (0.98, 1.48) | 1.30 (0.97, 1.75) | 0.83 (0.62, 1.12) | 1.11 (0.83, 1.48) | 1.20 (0.82, 1.77) | Tislelizumab |
| OS | | | | | |
| chemotherapy | 0.77 (0.62, 0.96) | 0.71 (0.57, 0.88) | 0.78 (0.63, 0.96) | 0.70 (0.52, 0.94) | 0.70 (0.57, 0.85) |
| 1.30 (1.04, 1.61) | Nivolumab | 0.92 (0.68, 1.25) | 1.01 (0.75, 1.37) | 0.91 (0.63, 1.31) | 0.91 (0.68, 1.22) |
| 1.41 (1.13, 1.75) | 1.08 (0.80, 1.48) | Camrelizumab | 1.10 (0.81, 1.49) | 0.99 (0.68, 1.43) | 0.98 (0.73, 1.33) |
| 1.28 (1.04, 1.58) | 0.99 (0.73, 1.34) | 0.91 (0.67, 1.23) | Pembrolizumab | 0.90 (0.63, 1.29) | 0.90 (0.67, 1.20) |
| 1.43 (1.06, 1.92) | 1.10(0.77, 1.58) | 1.02 (0.70, 1.46) | 1.12 (0.77, 1.60) | sintilimab | 1.00 (0.70, 1.43) |
| 1.43 (1.17, 1.75) | 1.10 (0.82, 1.48) | 1.02 (0.75, 1.36) | 1.12 (0.83, 1.49) | 1 .00(0.70, 1.43) | Tislelizumab |

*HR, hazard ratio; PFS, progression-free survival; OS, overall survival; CL, Confidence interval*

**eTable 3 PRISMA 2020 Checklist**

| **Section and Topic** | **Item #** | **Checklist item** | **Location where item is reported** |
| --- | --- | --- | --- |
| **TITLE** | | |  |
| Title | 1 | Identify the report as a systematic review. | Page 1 |
| **ABSTRACT** | | |  |
| Abstract | 2 | See the PRISMA 2020 for Abstracts checklist. | Page 3 |
| **INTRODUCTION** | | |  |
| Rationale | 3 | Describe the rationale for the review in the context of existing knowledge. | Page 7 |
| Objectives | 4 | Provide an explicit statement of the objective(s) or question(s) the review addresses. | Page 7 |
| **METHODS** | | |  |
| Eligibility criteria | 5 | Specify the inclusion and exclusion criteria for the review and how studies were grouped for the syntheses. | Page 7 |
| Information sources | 6 | Specify all databases, registers, websites, organisations, reference lists and other sources searched or consulted to identify studies. Specify the date when each source was last searched or consulted. | Page 7 |
| Search strategy | 7 | Present the full search strategies for all databases, registers and websites, including any filters and limits used. | Page 7 |
| Selection process | 8 | Specify the methods used to decide whether a study met the inclusion criteria of the review, including how many reviewers screened each record and each report retrieved, whether they worked independently, and if applicable, details of automation tools used in the process. | Page 7 |
| Data collection process | 9 | Specify the methods used to collect data from reports, including how many reviewers collected data from each report, whether they worked independently, any processes for obtaining or confirming data from study investigators, and if applicable, details of automation tools used in the process. | Page 7 |
| Data items | 10a | List and define all outcomes for which data were sought. Specify whether all results that were compatible with each outcome domain in each study were sought (e.g. for all measures, time points, analyses), and if not, the methods used to decide which results to collect. | Page 8 |
|  | 10b | List and define all other variables for which data were sought (e.g. participant and intervention characteristics, funding sources). Describe any assumptions made about any missing or unclear information. | Page 8 |
| Study risk of bias assessment | 11 | Specify the methods used to assess risk of bias in the included studies, including details of the tool(s) used, how many reviewers assessed each study and whether they worked independently, and if applicable, details of automation tools used in the process. | Page 8 |
| Effect measures | 12 | Specify for each outcome the effect measure(s) (e.g. risk ratio, mean difference) used in the synthesis or presentation of results. | Page 8 |
| Synthesis methods | 13a | Describe the processes used to decide which studies were eligible for each synthesis (e.g. tabulating the study intervention characteristics and comparing against the planned groups for each synthesis (item #5)). | Page 8 |
|  | 13b | Describe any methods required to prepare the data for presentation or synthesis, such as handling of missing summary statistics, or data conversions. | Page 8 |
|  | 13c | Describe any methods used to tabulate or visually display results of individual studies and syntheses. | Page 8 |
|  | 13d | Describe any methods used to synthesize results and provide a rationale for the choice(s). If meta-analysis was performed, describe the model(s), method(s) to identify the presence and extent of statistical heterogeneity, and software package(s) used. | Page 8 |
|  | 13e | Describe any methods used to explore possible causes of heterogeneity among study results (e.g. subgroup analysis, meta-regression). | Page 8 |
|  | 13f | Describe any sensitivity analyses conducted to assess robustness of the synthesized results. | Not applicable |
| Reporting bias assessment | 14 | Describe any methods used to assess risk of bias due to missing results in a synthesis (arising from reporting biases). | Not applicable |
| Certainty assessment | 15 | Describe any methods used to assess certainty (or confidence) in the body of evidence for an outcome. | Not applicable |
| **RESULTS** | | |  |
| Study selection | 16a | Describe the results of the search and selection process, from the number of records identified in the search to the number of studies included in the review, ideally using a flow diagram. | eFigure 1 |
|  | 16b | Cite studies that might appear to meet the inclusion criteria, but which were excluded, and explain why they were excluded. | Not applicable |
| Study characteristics | 17 | Cite each included study and present its characteristics. | eTable 1 |
| Risk of bias in studies | 18 | Present assessments of risk of bias for each included study. | eFigure 2 |
| Results of individual studies | 19 | For all outcomes, present, for each study: (a) summary statistics for each group (where appropriate) and (b) an effect estimate and its precision (e.g. confidence/credible interval), ideally using structured tables or plots. | Page 12 |
| Results of syntheses | 20a | For each synthesis, briefly summarise the characteristics and risk of bias among contributing studies. | Page 12 |
|  | 20b | Present results of all statistical syntheses conducted. If meta-analysis was done, present for each the summary estimate and its precision (e.g. confidence/credible interval) and measures of statistical heterogeneity. If comparing groups, describe the direction of the effect. | eTable 1  eFigure 3 |
|  | 20c | Present results of all investigations of possible causes of heterogeneity among study results. | Not applicable |
|  | 20d | Present results of all sensitivity analyses conducted to assess the robustness of the synthesized results. | Not applicable |
| Reporting biases | 21 | Present assessments of risk of bias due to missing results (arising from reporting biases) for each synthesis assessed. | Not applicable |
| Certainty of evidence | 22 | Present assessments of certainty (or confidence) in the body of evidence for each outcome assessed. | Not applicable |
| **DISCUSSION** | | |  |
| Discussion | 23a | Provide a general interpretation of the results in the context of other evidence. | Page 16 |
|  | 23b | Discuss any limitations of the evidence included in the review. | Page 18 |
|  | 23c | Discuss any limitations of the review processes used. | Page 18 |
|  | 23d | Discuss implications of the results for practice, policy, and future research. | Not applicable |
| **OTHER INFORMATION** | | |  |
| Registration and protocol | 24a | Provide registration information for the review, including register name and registration number, or state that the review was not registered. | Not applicable |
|  | 24b | Indicate where the review protocol can be accessed, or state that a protocol was not prepared. | Not applicable |
|  | 24c | Describe and explain any amendments to information provided at registration or in the protocol. | Not applicable |
| Support | 25 | Describe sources of financial or non-financial support for the review, and the role of the funders or sponsors in the review. | Page 20 |
| Competing interests | 26 | Declare any competing interests of review authors. | Page 21 |
| Availability of data, code and other materials | 27 | Report which of the following are publicly available and where they can be found: template data collection forms; data extracted from included studies; data used for all analyses; analytic code; any other materials used in the review. | Page 21 |

| **eTable 4. CHEERS 2022 Checklist** | | | |
| --- | --- | --- | --- |
|  | **Item** | **Guidance for Reporting** | **Reported in section** |
| **TITLE** | | |  |
| Title | 1 | Identify the study as an economic evaluation and specify the interventions being compared. | page 1 |
| **ABSTRACT** | | |  |
| Abstract | 2 | Provide a structured summary that highlights context, key methods, results and alternative analyses. | page 3-5 |
| **INTRODUCTION** | | |  |
| Background and objectives | 3 | Give the context for the study, the study question and its practical relevance for decision making in policy or practice. | page 5-7 |
| **METHODS** | | |  |
| Health economic analysis plan | 4 | Indicate whether a health economic analysis plan was developed and where available. | Page 9 |
| Study population | 5 | Describe characteristics of the study population (such as age range, demographics, socioeconomic, or clinical characteristics). | page 8 |
| Setting and location | 6 | Provide relevant contextual information that may influence findings. | page 7 |
| Comparators | 7 | Describe the interventions or strategies being compared and why chosen. | Page 8 |
| Perspective | 8 | State the perspective(s) adopted by the study and why chosen. | page 7 |
| Time horizon | 9 | State the time horizon for the study and why appropriate. | page 9 |
| Discount rate | 10 | Report the discount rate(s) and reason chosen. | page 9 |
| Selection of outcomes | 11 | Describe what outcomes were used as the measure(s) of benefit(s) and harm(s). | page 9 |
| Measurement of outcomes | 12 | Describe how outcomes used to capture benefit(s) and harm(s) were measured. | Page 9 |
| Valuation of outcomes | 13 | Describe the population and methods used to measure and value outcomes. | Page 9 |
| Measurement and valuation of resources and costs | 14 | Describe how costs were valued. | page 10-11 |
| Currency, price date, and conversion | 15 | Report the dates of the estimated resource quantities and unit costs, plus the currency and year of conversion. | Table 1 |
| Rationale and description of model | 16 | If modelling is used, describe in detail and why used. Report if the model is publicly available and where it can be accessed. | page 9 |
| Analytics and assumptions | 17 | Describe any methods for analysing or statistically transforming data, any extrapolation methods, and approaches for validating any model used. | page 9 |
| Characterizing heterogeneity | 18 | Describe any methods used for estimating how the results of the study vary for sub-groups. | Not applicable |
| Characterizing distributional effects | 19 | Describe how impacts are distributed across different individuals or adjustments made to reflect priority populations. | Table 1 |
| Characterizing uncertainty | 20 | Describe methods to characterize any sources of uncertainty in the analysis. | page 12 |
| Approach to engagement with patients and others affected by the study | 21 | Describe any approaches to engage patients or service recipients, the general public, communities, or stakeholders (e.g., clinicians or payers) in the design of the study. | Not applicable |
| **RESULTS** | | |  |
| Study parameters | 22 | Report all analytic inputs (e.g., values, ranges, references) including uncertainty or distributional assumptions. | Page 13 |
| Summary of main results | 23 | Report the mean values for the main categories of costs and outcomes of interest and summarise them in the most appropriate overall measure. | Table 2 |
| Effect of uncertainty | 24 | Describe how uncertainty about analytic judgments, inputs, or projections affect findings. Report the effect of choice of discount rate and time horizon, if applicable. | Page 14, Figure 2 |
| Effect of engagement with patients and others affected by the study | 25 | Report on any difference patient/service recipient, general public, community, or stakeholder involvement made to the approach or findings of the study | Not applicable |
| **DISCUSSION** | | |  |
| Study findings, limitations, generalizability, and current knowledge | 26 | Report key findings, limitations, ethical or equity considerations not captured, and how these could impact patients, policy, or practice. | page 15-19 |
| **OTHER RELEVANT INFORMATION** | | | |
| Source of funding | 27 | Describe how the study was funded and any role of the funder in the identification, design, conduct, and reporting of the analysis | page 23 |
| Conflicts of interest | 28 | Report authors conflicts of interest according to journal or International Committee of Medical Journal Editors requirements. | page 24 |
|  |  |  |  |
|  | | | |
